# Ischemia/Reperfusion-Induced MKP-3 Impairs Endothelial NO Formation via Inactivation of ERK1/2 Pathway

**DOI:** 10.1371/journal.pone.0042076

**Published:** 2012-07-27

**Authors:** Dan Yang, Ping Xie, Zhihua Liu

**Affiliations:** Institute of Medicinal Plant Development, Chinese Academy of Medical Sciences & Peking Union Medical College, Beijing, China; Goethe University, Germany

## Abstract

Mitogen-activated protein kinase phosphatases (MKPs) are a family of dual-specificity phosphatases. Endothelial cells express multiple MKP family members, such as MKP-3. However, the effects of MKP-3 on endothelial biological processes have not yet been fully elucidated. Here, we address the association between MKP-3 and endothelial Nitric oxide (NO) formation under ischemia/reperfusion (IS/RP) condition. Human umbilical vein endothelial cells (HUVECs) were subjected to IS/RP treatment. The MKP-3 expression and NO formation were examined. IS/RP induced endothelial MKP-3 expression and inhibited eNOS expression and NO formation, accompanied by an increase of endothelial apoptosis. The siRNA experiments showed that MKP-3 was an important mediator in impairing eNOS expression and NO production in endothelial cells. Transfection of HUVECs with constitutively active ERK plasmids suggested that the above mentioned effect of MKP-3 was via inactivation of ERK1/2 pathway. Furthermore, impairment of eNOS expression was restored by treatment of histone deacetylase (HDAC) inhibitor and related to histone deacetylation and recruitment of HDAC1 to the eNOS promoter. Finally, Salvianolic acid A (SalA) markedly attenuated induction of MKP-3 and inhibition of eNOS expression and NO formation under endothelial IS/RP condition. Overall, these results for the first time demonstrated that IS/RP inhibited eNOS expression by inactivation of ERK1/2 and recruitment of HDAC1 to the gene promoter, leading to decreased NO formation through a MKP-3-dependent mechanism in endothelial cells, and SalA has therapeutic significance in protecting endothelial cells from impaired NO formation in response to IS/RP.

## Introduction

Nitric oxide (NO) is a diatomic free-radical gas which is crucial for a variety of biological processes [1], [2]. NO is synthesized by a family of enzymes called NO synthases (NOS). There are three NOS isoforms in mammals: neuronal NOS (nNOS), endothelial NOS (eNOS), and inducible NOS (iNOS) [3]. Under physiological conditions, the dominant NOS isoform in the vasculature is eNOS, which catalyzes the formation of basal level NO to conduct physiological actions including effects on vascular smooth muscle cells, inhibition of adhesion of neutrophils and platelets to the endothelium, and maintenance of an anti-apoptotic environment in the vessel wall [4]. Rather than being a constitutive enzyme as was first suggested, eNOS is dynamically regulated at the transcriptional, posttranscriptional, and posttranslational levels [5]. Many physiopathological events, such as ischemia/reperfusion (IS/RP) injury, alter eNOS expression level or its activity, resulting in a change of NO formation [5]. Previous studies suggested that pleotropic signaling pathways were involved in regulation of eNOS activity, such as PI3K/AKT, MAPKs, and Rho-kinase [5]. However, the mechanisms by which IS/RP influence the eNOS activity are not clearly elucidated in endothelial cells.

Mitogen-activated protein kinase phosphatases (MKPs) are a family of dual-specificity phosphatases and play important roles in the regulation of p38, ERK1/2, and JNK signaling pathways which are induced by growth factors, cellular stress, and inflammatory cytokines [6], [7], [8], [9]. Endothelial cells express several MKPs, such as MKP-3 [9]. As a cytosolic phosphatase, MKP-3 prefers to target ERK1/2. To date, several studies demonstrated important roles of MKP-3 in endothelial apoptosis and leukocyte-endothelial adhesion [9], [10], [11]. However, no further investigations are provided concerning the effects of MKP-3 on other endothelial physiological and pathophysiological processes.

*S. miltiorrhiza*, commonly named “Danshen” in China, is a valuable medicinal plant [12], and can be used to promote blood flow and to resolve blood stasis. It is also an important part in compound danshen dripping pills which have been passed phase II human clinical trial of Food and Drug Administration (FDA). Salvianolic acid A, also known as SalA, is one of the main active, water-soluble components in *S. miltiorrhiza*. Previous studies have indicated the beneficial effects of SalA on preventing oxidative stress, platelet aggregation, ischemia, and hepatocirrhosis [13].

In our preliminary microarray data, we found that IS/RP upregulated MKP-3 expression, which was inhibited significantly by SalA pretreatment. Further, eNOS expression level showed opposite results after the same treatment method. Thus, in the present study, we firstly address the correlation between MKP-3 and eNOS expression under IS/RP condition in endothelial cells. Moreover, we detected whether SalA exhibits beneficial effects in these processes.

## Results

### The Expression Level of MKP Family Members and eNOS in Microarray Data

In our preliminary study, HUVECs were subjected to IS/RP injury with or without SalA pretreatment, and then the expression profile was obtained through microarray technique. The data demonstrated that IS/RP resulted in enhanced expression of MKP family members, such as MKP-3, MKP-5, and MKP-7, and interestingly, SalA merely inhibited IS/RP-upregulated MKP-3 expression. Furthermore, eNOS expression was also changed after IS/RP with or without SalA pretreatment, and presented an opposite results in contrast to MKP-3 (*Table 1*). These data indicated that maybe a negative correlation existed between MKP-3 and eNOS under IS/RP condition.

**Table 1:** The changed expression levels of MKP family members and eNOS after IS/RP and SalA treatment.

Gene	Synonym	Fold change	
		IS/RP vs. Control	(IS/RP+SalA) vs. IS/RP
DUSP1	MKP-1	1.51	0.98
DUSP4	MKP-2	2.3*	1.11
DUSP5	B23, hVH3	2.46*	1.24
DUSP6	MKP-3	4.11*	0.32#
DUSP10	MKP-5	1.6*	0.97
DUSP16	MKP-7	1.54	1.01
NOS3	eNOS	0.41*	1.98#

* *P*<0.05, IS/RP group vs. untreated control group;

# *P*<0.05, SalA-treated IS/RP group vs. IS/RP group.

### IS/RP Induces Endothelial MKP-3 Expression

**Figure 1:**
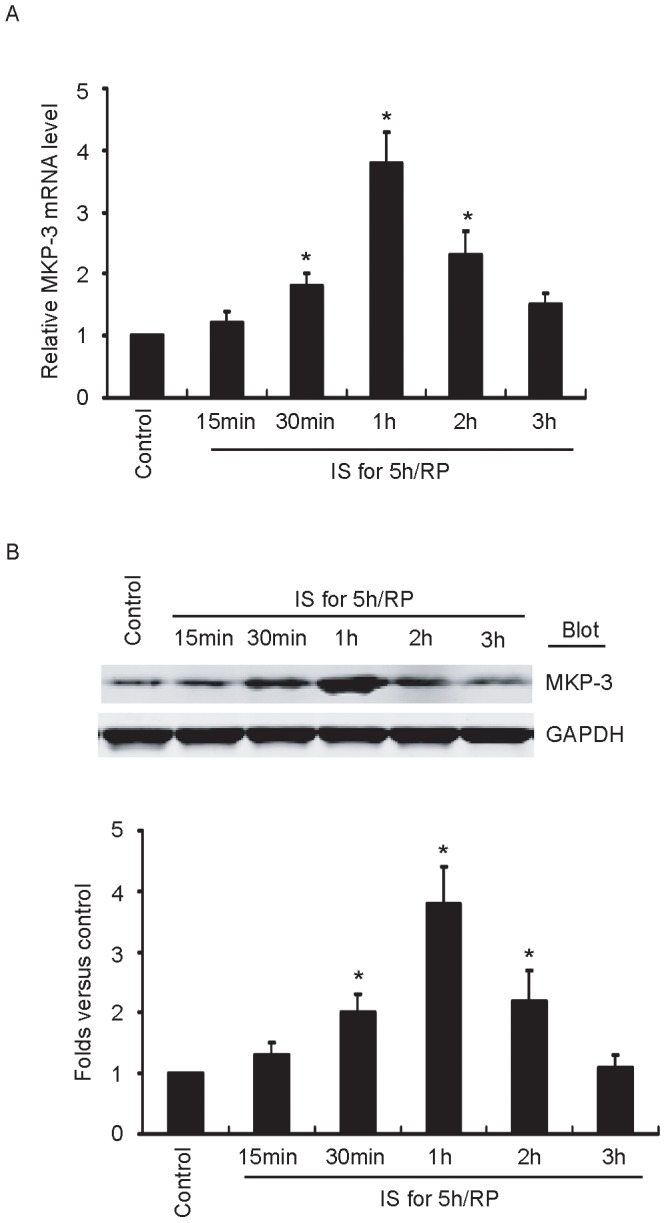
IS/RP induces endothelial MKP-3 expression. HUVECs were subjected to 5 hours ischemia followed by reperfusion for indicated time points. (a) The MKP-3 mRNA levels in each group were detected by quantitative RT-PCR (n = 5, means±SEM). *P<0.05 vs. untreated group (control). (b) The protein levels of MKP-3 and GAPDH were detected by immunoblot analysis using indicated antibodies. A representative blot (top) was shown (n = 4). Quantitative analysis of MKP-3 (bottom) was expressed as fold increase above control group (n = 4, means±SEM). *P<0.05 vs. control group.

Firstly, we investigated whether IS/RP induced endothelial MKP-3 expression. Cells were treated with ischemia for 5 hours and reperfusion for indicated time points, and then MKP-3 expression level was detected. As shown in Figure 1a, IS/RP led to a significant increase in MKP-3 mRNA level, which reached a peak about four-fold increase at 1 hour reperfusion and declined to near basal level at 3 hours. Additionally, the expression pattern of MKP-3 protein was similar to that of mRNA (Fig. 1b).

### IS/RP Inhibits eNOS Expression and Endothelial NO Formation

**Figure 2:**
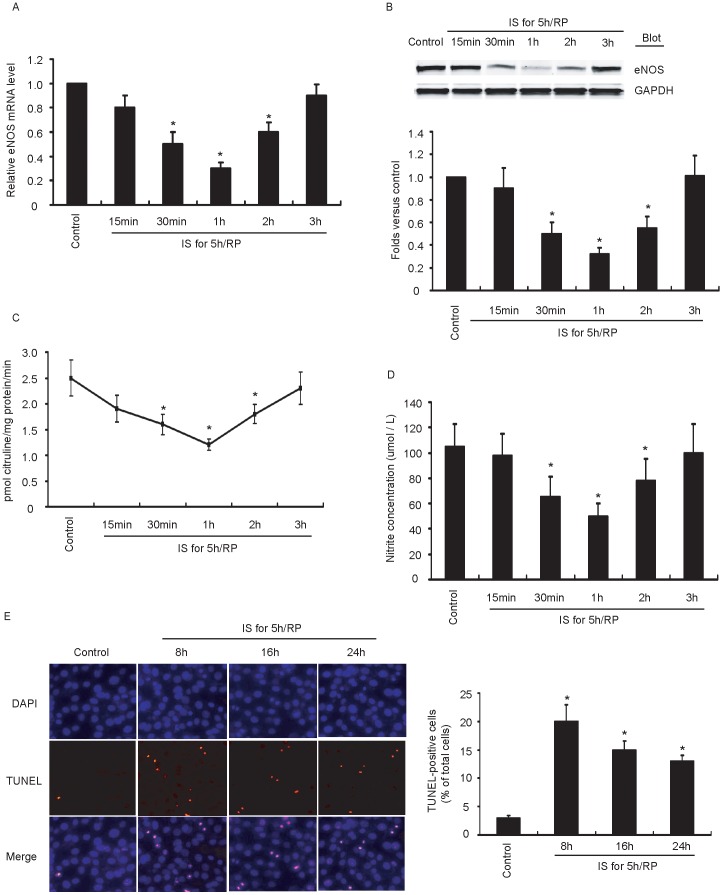
IS/RP inhibits eNOS expression and NO formation. HUVECs were subjected to IS/RP treatment. (a) The eNOS mRNA levels in each group were detected by quantitative RT-PCR (n = 5, means±SEM). *P<0.05 vs. untreated group (control). (b) The protein levels of eNOS and GAPDH were detected by immunoblot analysis using indicated antibodies. A representative blot (top) was shown (n = 4). Quantitative analysis of eNOS (bottom) was expressed as fold increase above control group (n = 4, means±SEM). *P<0.05 vs. control group. (c) The NOS activity in the cell lysates was determined via examining the efficiency in conversion of [3H]L-arginine to [3H]L-citrulline (n = 4, means±SEM). *P<0.05 vs. untreated control group. (d) NO formation was detected as nitrite released into supernatant of confluent cultures of HUVECs (see Methods) (n = 4, means±SEM). *P<0.05 vs. untreated control group. (e) Cell apoptosis was determined by TUNEL assay. A representative field (left) was shown for each condition (magnification, ×200). The number of TUNEL-positive cells was quantified, and at least 120 cells per dish were counted (n = 3, means ± SEM.). *P<0.05 vs. control group.

It is reported that NO production is altered after IS/RP injury in cardiovascular system [14], [15]. We next investigated whether IS/RP influenced eNOS expression and NO formation in endothelial cells. After treatment of cells with IS/RP, we observed significant decrease in eNOS mRNA level and protein level (Fig. 2a, b). It is known that eNOS expression does not always correlate with eNOS activity, so we further detected eNOS activity and endothelial NO production. As shown in Figure 2c and d, eNOS activity and NO formation reduced markedly in response to IS/RP, correlating well with alteration of eNOS expression pattern. The eNOS-derived NO maintains an anti-apoptotic environment in vessel wall. We observed increased endothelial apoptosis accompanying inhibited NO formation (Fig. 2e). Thus, these results suggested that IS/RP impaired eNOS expression, leading to decreased NO production as well as enhanced apoptosis in endothelial cells.

### IS/RP-induced MKP-3 Impairs eNOS Expression and Endothelial NO Formation

**Figure 3:**
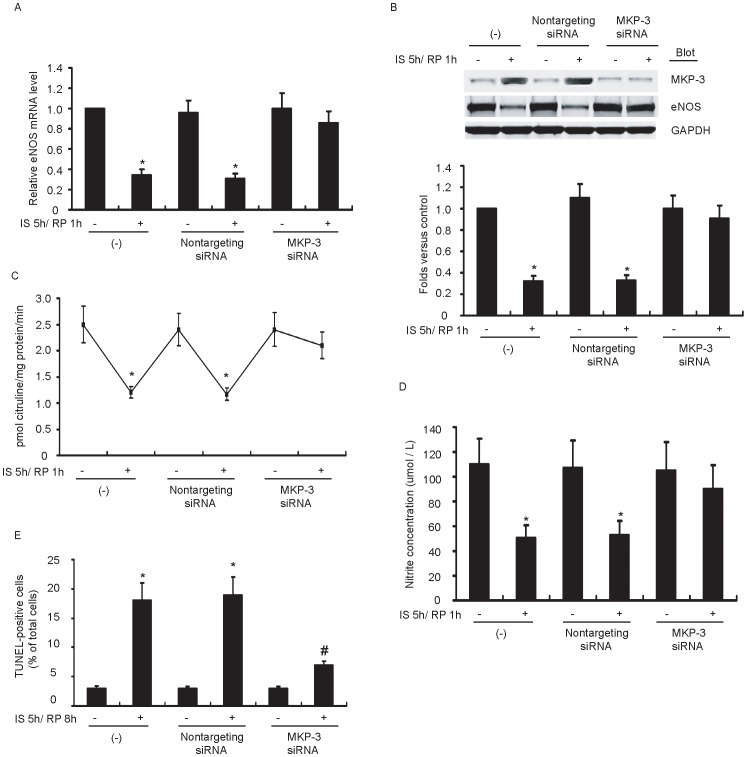
IS/RP-induced MKP-3 impairs eNOS expression and NO formation. HUVECs were transfected with MKP-3 siRNA (50 nmol/L) or nontargeting siRNA (50 nmol/L). After 48 h, cells were normally cultured or subjected to IS/RP treatment. (a) The eNOS mRNA levels in each group were detected by quantitative RT-PCR (n = 5, means±SEM). *P<0.05 vs. parallel normally cultured cells. (b) The protein levels of MKP-3, eNOS, and GAPDH were detected by immunoblot analysis using indicated antibodies. A representative blot (top) was shown (n = 4). Quantitative analysis of eNOS (bottom) was expressed as fold increase above control group (nontransfected and normally cultured cells) (n = 4, means±SEM). *P<0.05 vs. parallel normally cultured cells. (c) The NOS activity in the cell lysates was determined via examining the efficiency in conversion of [3H]L-arginine to [3H]L-citrulline (n = 4, means±SEM). *P<0.05 vs. parallel normally cultured cells. (d) NO formation was detected as nitrite released into supernatant of confluent cultures of HUVECs (n = 4, means±SEM). *P<0.05 vs. parallel normally cultured cells. (e) Cell apoptosis was determined by TUNEL assay. The number of TUNEL-positive cells was quantified (n = 3, means ± SEM.). *P<0.05 vs. control group; ^#^ *P*<0.05 vs. nontargeting siRNA-transfected IS/RP group.

To further determine whether there is a relationship between IS/RP-induced MKP-3 and impaired eNOS expression and NO formation, we used the siRNA technique to suppress MKP-3 expression in HUVECs. We first optimized the conditions for cell transfection and tested MKP-3 expression. A significant reduction of MKP-3 expression was observed in cells transfected with MKP-3 siRNA compared with nontargeting siRNA (data not shown). MKP-3 siRNA markedly attenuated IS/RP-mediated reduction of eNOS expression level (Fig. 3a, b). and activity (Fig. 3c) as well as NO production (Fig. 3d). Furthermore, endothelial apoptosis were partially reversed by MKP-3 siRNA treatment (Fig. 3e). Additionally, nontargeting siRNA had no effect on these processes (Fig. 3c–e). Thus, these results provided a causal relationship between IS/RP-mediated induction of MKP-3 and inhibition of eNOS expression and NO formation in endothelial cells.

### IS/RP-induced MKP-3 Leads to Dephophorylation of ERK1/2

**Figure 4:**
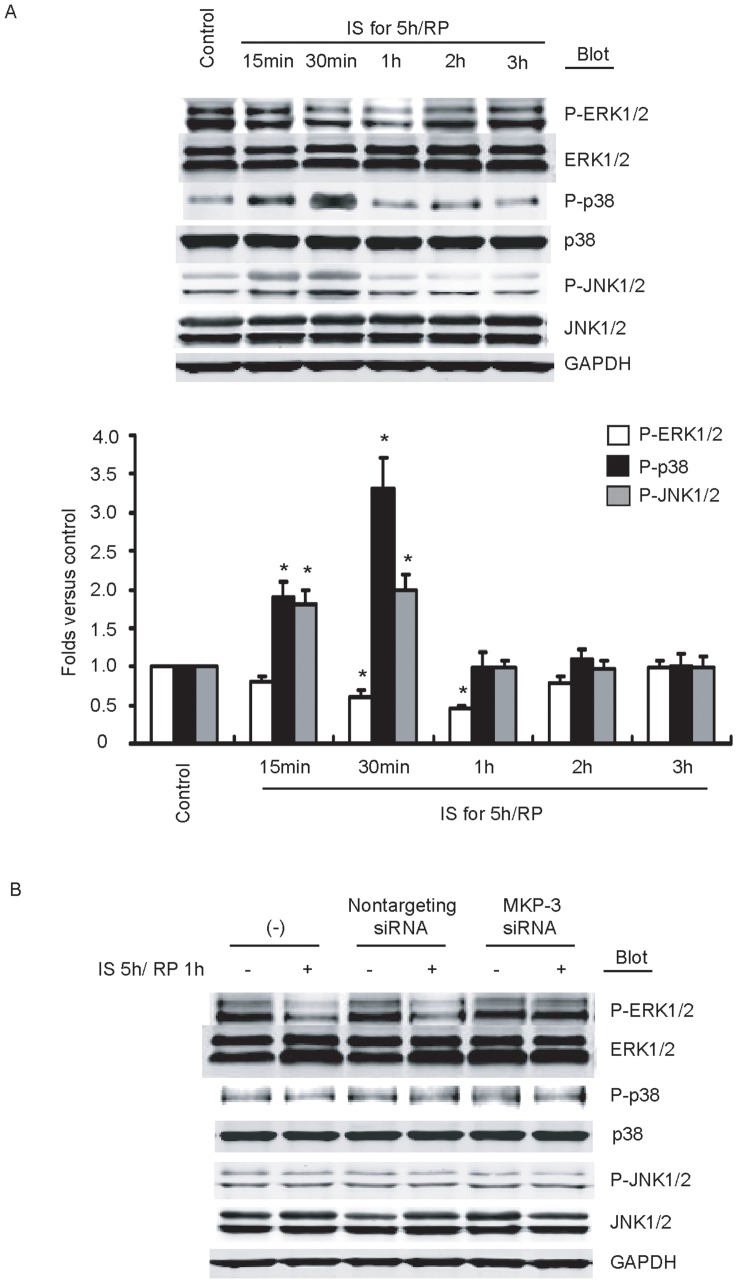
IS/RP-induced MKP-3 leads to dephophorylation of ERK1/2. (a) HUVECs were subjected to IS/RP treatment. The protein levels of ERK1/2, p38, JNK1/2, and GAPDH were detected by immunoblot analysis using indicated antibodies. A representative blot (top) was shown (n = 4). Quantitative analysis of ERK1/2, p38, and JNK1/2 (bottom) was expressed as fold increase above control group (n = 4, means±SEM). *P<0.05 vs. control group. (b) HUVECs were transfected with MKP-3 siRNA (50 nmol/L) or nontargeting siRNA (50 nmol/L). After 48 h, cells were normally cultured or subjected to IS/RP treatment. The protein levels of ERK1/2, p38, JNK1/2, and GAPDH were detected by immunoblot analysis using indicated antibodies. A representative blot was shown (n = 4).

To determine the precise mechanism by which MKP-3 inhibits eNOS expression and NO production, the action of MKP-3 on its potential downstream kinases p38, JNK1/2, and ERK1/2 was examined. Firstly, we detected whether IS/RP altered endothelial p38, JNK1/2, and ERK1/2 pathways. IS/RP caused a marked dephosphorylation of ERK1/2 (Fig. 4a). Moreover, reperfusion for 30 minutes resulted in activation of p38 and JNK1/2 (Fig. 4a), which was in accordance with previous study [16]. Secondly, we used the siRNA technique to suppress MKP-3 expression to detect whether MKP-3 influences the phosphorylation levels of p38, JNK1/2, and ERK1/2. As shown in Figure 4b, MKP-3 siRNA markedly abolished IS/RP-mediated inhibition of ERK1/2 activation, whereas no effect was observed of MKP-3 on phosphorylation levels of p38 and JNK1/2. Additionally, the early activation of p38 and JNK1/2 induced by IS/RP was not affected by MKP-3 siRNA as well (data not shown). Together, these results indicated that IS/RP-induced MKP-3 led to dephophorylation of ERK1/2 in endothelial cells, without effect on p38 and JNK1/2 activation.

### IS/RP-induced MKP-3 Impairs eNOS Expression and Endothelial NO Formation via Inactivation of ERK1/2 Pathway

**Figure 5:**
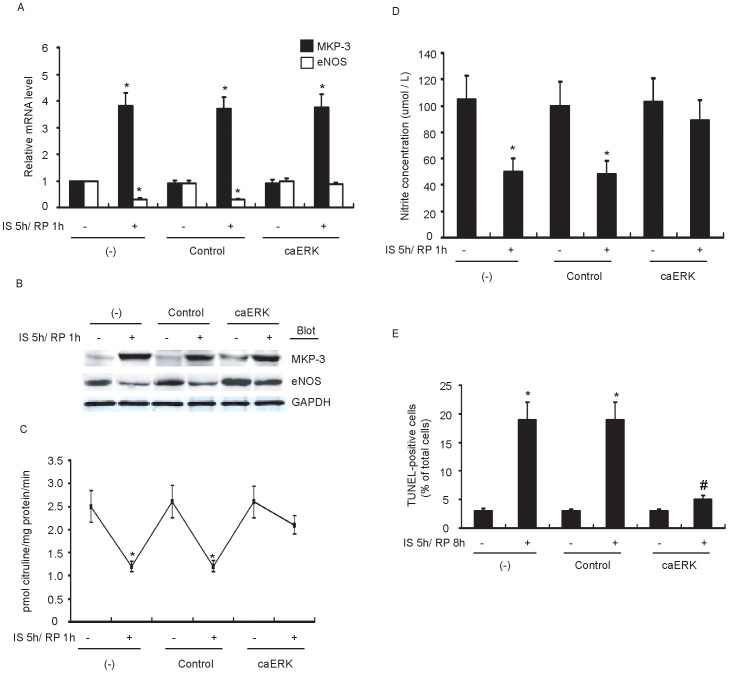
IS/RP-induced MKP-3 impairs eNOS expression and NO formation via inactivation of ERK1/2 pathway. HUVECs were not transfected (−) or transfected with control plasmids (control) or constitutively active HA-ERK1 and c-myc-ERK2 expression plasmids (caERK). After 48 h, cells were normally cultured or subjected to IS/RP treatment. (a) The MKP-3 and eNOS mRNA levels in each group were detected by quantitative RT-PCR (n = 5, means±SEM). *P<0.05 vs. parallel normally cultured cells. (b) The protein levels of MKP-3, eNOS, and GAPDH were detected by immunoblot analysis. A representative blot was shown (n = 4). (c) The NOS activity in the cell lysates was determined via examining the efficiency in conversion of [3H]L-arginine to [3H]L-citrulline (n = 4, means±SEM). *P<0.05 vs. parallel normally cultured cells. (d) NO formation was detected as nitrite released into supernatant of confluent cultures of HUVECs (n = 4, means±SEM). *P<0.05 vs. parallel normally cultured cells. (e) Cell apoptosis was determined by TUNEL assay. The number of TUNEL-positive cells was quantified (n = 3, means ± SEM.). *P<0.05 vs. control group; ^#^ *P*<0.05 vs. IS/RP group of transfection control.

We next examined whether the inhibitory effect of MKP-3 on ERK1/2 activation led to impaired eNOS expression and NO production. We transfected HUVECs with constitutively active ERK1/2 expression plasmids (caERK), and noticed a transfection efficiency up to 70% as determined using green fluorescent protein. The results showed that caERK transfection significantly attenuated the impairment of eNOS expression (Fig. 5a, b) and activity (Fig. 5c), as well as NO formation (Fig. 5d). Moreover, endothelial apoptosis were partially reversed by caERK transfection as well (Fig. 5e). However, MKP-3 expression level was not altered (Fig. 5a, b). Additionally, we studied the roles of p38 and JNK1/2 in eNOS expression as well, and detected no obvious changes after treatment of specific inhibitors of p38 and JNK1/2 (SB203580 and SP600125) (data not shown). Taken together, these results demonstrated that IS/RP-mediated inhibition of eNOS expression and NO formation by MKP-3 was through inactivation of ERK1/2 pathway.

### The Impaired eNOS Expression is Mediated by HDAC1 Binding to the Gene Promoter

**Figure 6:**
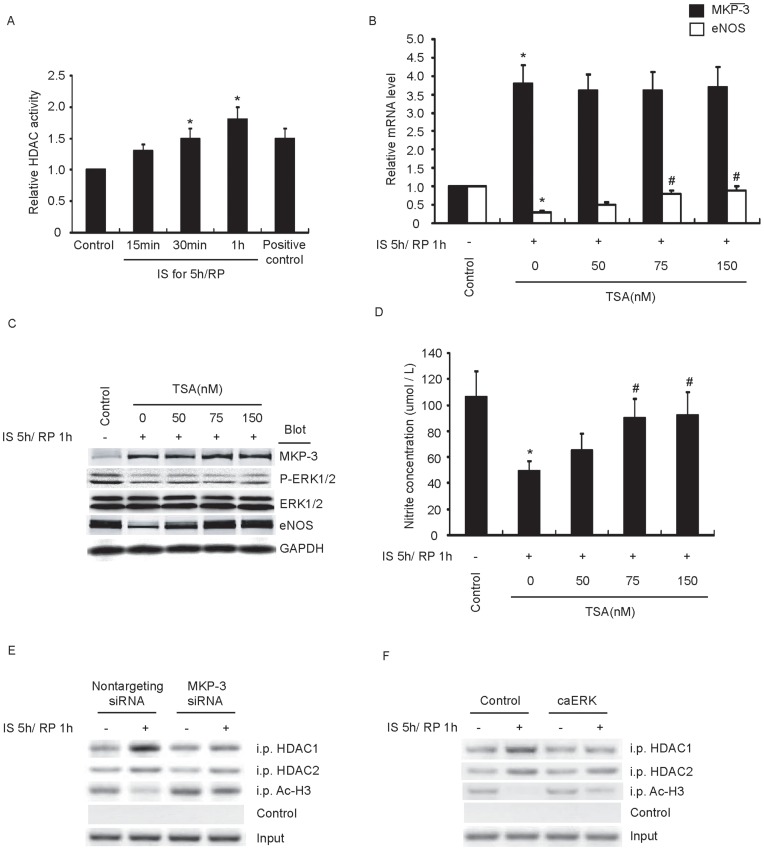
The impaired eNOS expression is mediated by histone deacetylation at gene promoter. (a) HUVECs were subjected to IS/RP treatment and 10µg of nuclear extract in each group was assayed for HDAC enzyme activity (n = 4, means±SEM). *P<0.05 vs. untreated group (control). (b) HUVECs were pretreated with TSA in various concentrations, and then subjected to IS/RP treatment. The mRNA levels of MKP-3 and eNOS in each group were detected by quantitative RT-PCR (n = 4, means±SEM). *P<0.05 vs. control group; ^#^ *P*<0.05 vs. non-TSA-treated IS/RP group. (c) The protein levels of MKP-3, ERK1/2, eNOS, and GAPDH were detected by immunoblot analysis. A representative blot was shown (n = 3). (d) NO formation was detected (n = 3, means±SEM). *P<0.05 vs. control group; ^#^ *P*<0.05 vs. non-TSA-treated IS/RP group. (e) HUVECs were transfected with MKP-3 siRNA (50 nmol/L) or nontargeting siRNA (50 nmol/L). After 48 h, cells were normally cultured or subjected to IS/RP treatment. Cells were then analyzed using the ChIP assay with antibodies Ac-H3 or HDAC1 or HDAC2 (see Method). Figure is representative of three separate experiments. (f) HUVECs were transfected with control plasmids (control) or constitutively active HA-ERK1 and c-myc-ERK2 expression plasmids (caERK). After 48 h, cells were normally cultured or subjected to IS/RP treatment. Cells were then analyzed using the ChIP assay. Figure is representative of three separate experiments.

Histone acetylation modification participates in transcriptional regulation of genes by affecting the status of chromatin remodeling. Histone deacetylases (HDACs) catalyze the removal of acetyl groups from histones and contribute to transcriptional repression. Here, we studied whether the down-regulation of eNOS expression by MKP-3 was concerned in histone deacetylation and recruitment of HDACs to the eNOS promoter. Firstly, we noticed that global HDAC activity was increased in endothelial cells by IS/RP treatment (Fig. 6a). HDAC inhibitor Trichostatin (TSA) significantly inhibited IS/RP-impaired eNOS expression in a dose-dependent manner (Fig. 6b, c), and attenuation of impaired NO formation was also observed (Fig. 6d). These results suggested that HDACs maybe played roles in IS/RP-mediated down-regulation of eNOS expression. Thus, we further detected whether the binding of HDACs to eNOS promoter was changed. ChIP assays showed that IS/RP increased the recruitment of HDAC1 to the promoter of eNOS, and this process was hampered significantly by transfection of MKP-3 siRNA or caERK (Fig. 6e, f). However, we detected slight alteration concerning the binding of HDAC2 to eNOS promoter (Fig. 6e, f). Additionally, acetylated histone H3 (Ac-H3) was regulated in an opposing manner compared with HDAC1 (Fig. 6e, f). Overall, these studies implicated that the down-regulation of eNOS expression via MKP-3/ERK1/2 signaling was associated with histone deacetylation and recruitment of HDAC1 to the eNOS promoter.

### SalA Alleviates Impairment of Endothelial NO Formation in Response to IS/RP

**Figure 7:**
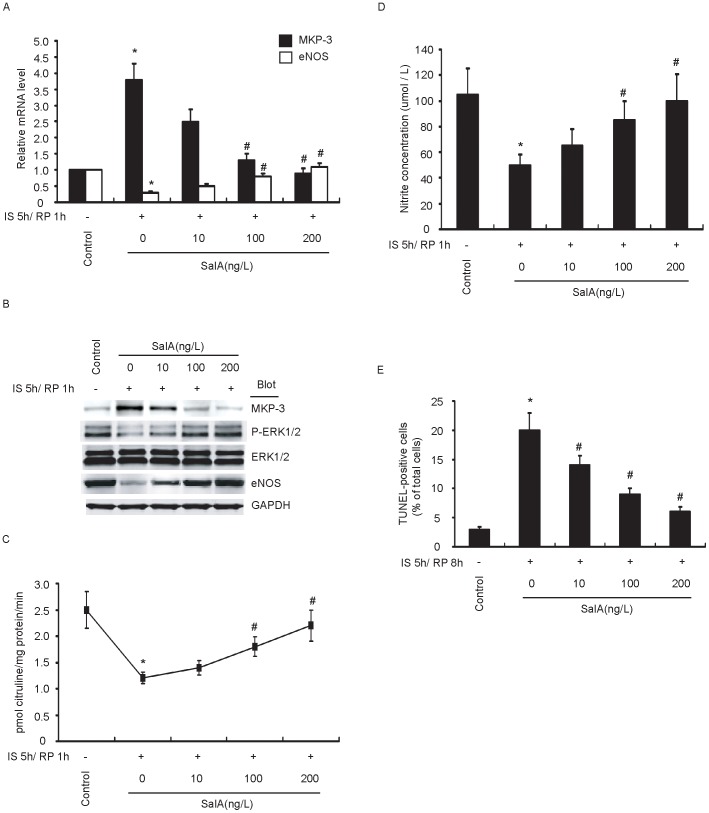
SalA alleviates impairment of endothelial NO formation in response to IS/RP. HUVECs were pretreated with SalA for 30 min in various concentrations, and then subjected to IS/RP treatment. (a) The MKP-3 and eNOS mRNA levels in each group were detected by quantitative RT-PCR (n = 5, means±SEM). *P<0.05 vs. control group; ^#^ *P*<0.05 vs. non-SalA-treated IS/RP group. (b) The protein levels of MKP-3, ERK1/2, eNOS, and GAPDH were detected by immunoblot analysis. A representative blot was shown (n = 4). (c) The NOS activity in the cell lysates was determined via examining the efficiency in conversion of [3H]L-arginine to [3H]L-citrulline (n = 4, means±SEM). *P<0.05 vs. control group; ^#^ *P*<0.05 vs. non-SalA-treated IS/RP group. (d) NO formation was detected as nitrite released into supernatant of confluent cultures of HUVECs (n = 3, means±SEM). *P<0.05 vs. control group; ^#^ *P*<0.05 vs. non-SalA-treated IS/RP group. (e) Cell apoptosis was determined by TUNEL assay. The number of TUNEL-positive cells was quantified, and at least 120 cells per dish were counted (n = 3, means ± SEM.). *P<0.05 vs. control group; ^#^ *P*<0.05 vs. non-SalA-treated IS/RP group.

SalA is one of the main active, water-soluble components in *S. miltiorrhiza*, a valuable medicinal plant. Previous study has indicated the beneficial effects of SalA on preventing oxidative stress, platelet aggregation, ischemia, and hepatocirrhosis [13]. In our preliminary microarray data, we found that SalA abolished IS/RP-mediated induction of MKP-3 and inhibition of eNOS expression. Thus, we addressed the event that whether SalA exhibited beneficial effects on restoring IS/RP-impaired eNOS expression and NO formation. As shown in Figure 7a and b, SalA markedly abolished IS/RP-altered MKP-3 and eNOS expression level. Furthermore, SalA reversed IS/RP-impaired eNOS activity and NO formation (Fig. 7c, d), which was accompanied by attenuated endothelial apoptosis (Fig. 7e). In addition, all these actions of SalA were in a dose-dependent manner. Taken these results together, SalA was beneficial to inhibition of MKP-3 expression and restoration of impaired eNOS expression and NO production in endothelial IS/RP injury.

## Discussion

In the present study, we provided evidence for the first time that IS/RP-induced MKP-3 impaired eNOS expression and NO formation in endothelial cells, and the actions of MKP-3 were mediated through inactivation of ERK1/2 pathway and recruitment of HDAC1 to the eNOS promoter. Moreover, we found a potential drug component from a medicinal plant, SalA, which effectively attenuated IS/RP-mediated induction of MKP-3 and inhibition of eNOS expression and NO formation.

NO is a critical effective molecule in the cardiovascular system. Under physiological conditions, endothelial basal NO formation catalyzed by eNOS plays an important role in vascular homeostasis affecting vasomotor tone as well as vascular smooth muscle proliferation, platelet aggregation, expression of adhesion molecules, inhibition of lipid oxidation, and regulation of apoptosis [17], [18]. Numerous physiological and pathophysiological stimuli such as shear stress, hypoxia, and inflammatory mediators influence eNOS activity [19], [20]. Many studies have shown that IS/RP injury impairs eNOS activity leading to a decreased basal NO production [14], [21]. In our study, we found that eNOS expression level was markedly decreased after IS/RP with reduction of eNOS activity and NO formation as well as increase in endothelial apoptosis (Fig. 2). However, it should be taken into mind that decreased NO formation was not always due to inhibited eNOS expression. Other than at transcriptional level, eNOS activity can also be regulated at posttranscriptional levels via phosphorylation, S-nitrosylation, and interaction of eNOS with other proteins, such as caveolins [22], [23], [24]. Our results showed that decreased eNOS expression correlated well with inhibited eNOS activity and NO formation in the first hours of reperfusion (Fig. 2). Taken together, these results suggested that IS/RP mediated reduction in eNOS activity at transcriptional level leading to an inhibition of NO production in endothelial cells.

MKPs are a family of dual-specificity phosphatases and play important roles in the regulation of p38, ERK1/2, and JNK signaling pathways [6], [7], [8], [9]. However, not much is known currently about the normal physiological functions of many of the MKP family members. To date, the biological demonstration of MKPs are mainly focused on cancer disease [25], [26]. Endothelial cells express multiple MKP family members. In our preliminary microarray results, we observed altered expression levels of several MKP family members in response to IS/RP treatment, including MKP-1, -2, -3, -5, and -7. However, about the biological effect in IS/RP, all the MKPs have not yet been investigated except MKP-1. MKP-1 could suppress IS/RP-induced cell death possibly through regulating JNK signaling [16], [27]. Moreover, the mechanisms responsible for IS/RP-mediated induction of MKP-1 as well as other MKP members remain to be determined. MKP-3 is unique within the MKP family, because it is exclusively located in the cytosol, indicating a specific regulatory role in inactivating Mitogen-activated protein kinases by targeting cytoplasmic substrates or by blocking nuclear localization [28]. At present, several studies demonstrated important roles of MKP-3 in endothelial apoptosis and leukocyte-endothelial adhesion [9], [10], [11]. However, no further evidence is presented addressing the actions of MKP-3 on other endothelial physiological and pathophysiological events. Our current study showed that MKP-3 was induced under IS/RP condition (Fig. 1). Some study showed that MKP-3 expression has been linked to Ras activity in cancer cells [29], [30]. However, little is known presently about the mechanism involved in endothelial MKP-3 induction. Maybe the oxidative stress, increase in cytosolic Ca^2+^, or other IS/RP-related events responsible for this process.

It was noteworthy in our study that, after MKP-3 expression was suppressed effectively by siRNA technique, IS/RP-impaired eNOS expression, eNOS activity, and NO formation were largely abolished (Fig. 3a–d). Thus, these results indicated that there was a causal relationship between IS/RP-mediated induction of MKP-3 and inhibition of eNOS expression and NO formation, which, more or less, provided us a better understanding of the biological actions of MKP-3 in endothelial cells. Of the MKP family members, it is MKP-3 that specifically inactivates ERK1/2. In our study, we observed significant up-regulation of phosphorylated ERK1/2 after transfection of HUVECs with MKP-3 siRNA, while phosphorylated p38 and JNK1/2 levels were unchanged (Fig. 4b). Furthermore, after transfection of HUVECs with caERK plasmids, no obvious reduction of eNOS expression and NO formation was detected under IS/RP condition (Fig. 5a–d), indicating that inactivation of ERK1/2 contributed to decrease in endothelial NO production. Taken these results together, we speculated that IS/RP-induced MKP-3 dephosphorylated ERK1/2, leading to impaired basal levels of eNOS expression, and NO formation in endothelial cells.

The acetylation of core histones correlates with transcriptional activity in many genes, allowing DNA to unfold and providing access for transcription factors to bind to their targeted promoters. The turnover of acetylated histones is regulated by the opposing activities of histone acetyltransferases (HATs) and histone deacetylases (HDACs), where HATs generally allow transcription and HDACs repress transcription. HDACs were shown to be involved originally in cancer and neurological disease, and recent reports implicated their crucial roles in cardiovascular disease [31]. The broad inhibitor of HDACs, TSA, has been shown to influence eNOS expression in HUVECs [19], [32]. In line with this, we noticed that IS/RP enhanced global HDAC activity (Fig. 6a) and inhibition of HDACs by TSA alleviated IS/RP-impaired eNOS expression dose-dependently (Fig. 6b, c), suggesting crucial roles of HDACs in down-regulation of eNOS expression. Current studies reported that HDAC1 and 2 were related to cardiovascular system, which were widely expressed and resided nearly exclusively in the cell nucleus [19], [31]. HDAC1 was shown to be involved in regulaton of eNOS promoter activity [32], [33]. In our result, IS/RP increased the binding of HDAC1 to the promoter of eNOS, which was hampered significantly by transfection of MKP-3 siRNA or caERK (Fig. 6e, f). Accordingly, Ac-H3 was regulated in an opposing manner as compared with HDAC1 (Fig. 6e, f). However, it seemed that the alteration was not much obviously concerning the binding of HDAC2 to eNOS promoter (Fig. 6e, f). Thus, our findings indicated that histone deacetylation and recruitment of HDAC1 to the eNOS promoter might be implicated in the down-regulation of eNOS gene expression via MKP-3/ERK1/2 signaling.

SalA is one of the main active, water-soluble components in *S. miltiorrhiza*, which is commonly named “Danshen” in China, and has been widely used as a medicinal plant for more than two thousand years [12]. *S. miltiorrhiza* is an important part in compound danshen dripping pills which have a therapeutic potential on cardiac arrest treatment and have been passed phase II human clinical trial of FDA. In the past few years, many beneficial effects of SalA on cardiovascular system have been proposed [13]. Furthermore, mechanisms of how SalA regulate biological processes in endothelial cells, vascular smooth muscle cells, and cardiomyocytes have been investigated [34], [35], [36]. During IS/RP injury, SalA has been reported to be a cardiovascular protective agent, mainly through its anti-apoptosis activity via induction of Bcl-2 and inhibition of Bax [37], [38]. Further, anti-inflammatory property was suggested as well which was correlated with the inhibition of granulocyte adherence [39]. In the presented study, SalA was firstly provided as a potential protectant against IS/RP-impaired endothelial NO formation. We found that SalA blocked IS/RP-incuced MKP-3 expression dose-dependently (Fig. 7a, b), and interestingly, decreased phosphor-ERK1/2, eNOS expression, eNOS activity, and NO formation as well as endothelial apoptosis in response to IS/RP were also markedly attenuated by SalA treatment (Fig. 7a–e). Thus, SalA demonstrated effective protection against IS/RP-impaired NO production maybe through its inhibition of MKP-3 leading to restoration of hampered ERK1/2 activation and eNOS expression, which has therapeutic significance in the states of IS/RP-induced vasculature injuries.

**Figure 8:**
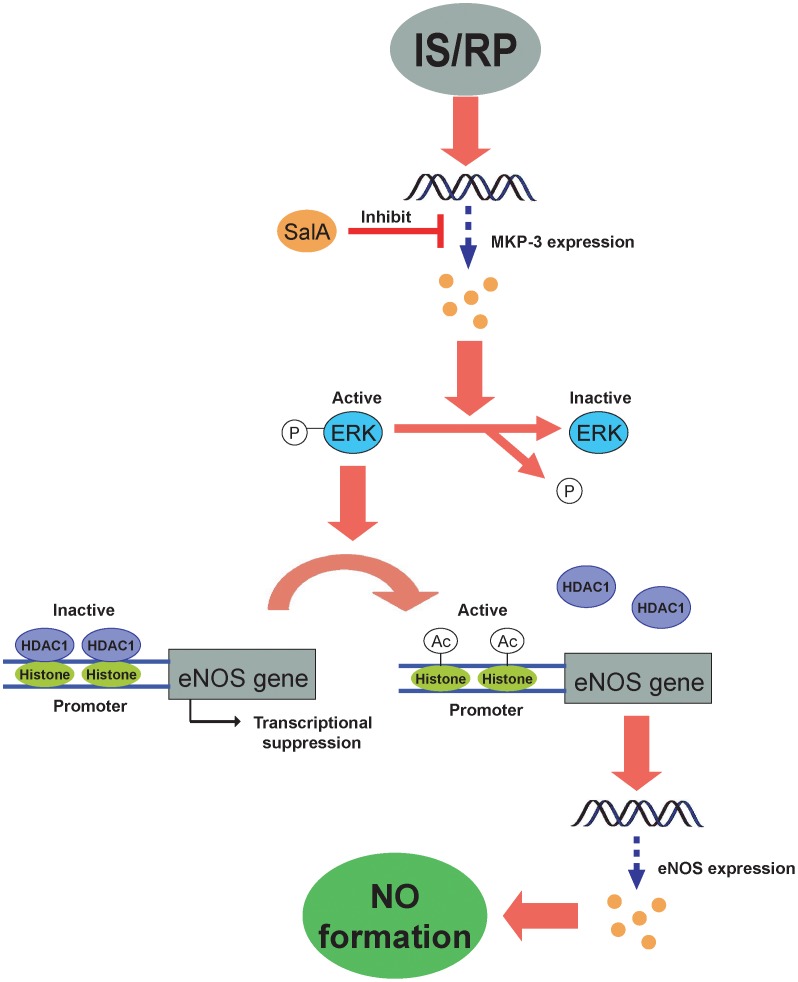
Proposed model of MKP-3/eNOS signaling. Ischemia/reperfusion (IS/RP) induces MKP-3 expression, leading to inactivation of ERK1/2 and recruitment of HDAC1 to the eNOS promoter, thus attenuation of eNOS expression and NO formation. Furthermore, SalA inhibits induced MKP-3 expression, protecting endothelial cells against impaired eNOS expression and NO formation in response to IS/RP injury.

In conclusion, our study demonstrated that IS/RP-induced MKP-3 impaired eNOS expression and NO formation via inactivation of ERK1/2 pathway and recruitment of HDAC1 to the eNOS promoter, which provided us not only a better understanding of the biological actions of MKP-3 in endothelial cells, but also a novel mechanism to explain impaired basal endothelial NO production in response to IS/RP. Moreover, we presented SalA as a potential drug component to effectively alleviate IS/RP-mediated induction of MKP-3 and inhibition of NO production (Fig. 8). Thus, these findings may have significance for the therapeutic application in IS/RP-related diseases.

## Materials and Methods

The study was reviewed and approved by the institutional ethics review board of the Peking Union Medical College (Beijing, China) and also conforms with the principles outlined in the Declaration of Helsinki (Cardiovasc Res 1997;35∶2–4). This work was supported by National Natural Science Foundation of China (81102746, 81100077), Beijing Natural Science Foundation (5113033), Scientific Research Foundation of the State Human Resource Ministry and the Education Ministry for Returned Chinese Scholars, New Star Project of Peking Union Medical College, Youth Foundation of Peking Union Medical College, the Research Fund for the Doctoral Program of Higher Education (20111106120028), “Major Drug Discovery” major science and technology research “12nd Five-Year Plan” (2012ZX09301-002-001), China Medical Board of New York (A2009001).

### Chemicals and Reagents

Antibodies used were as follows: anti-MKP-3, anti-eNOS, anti-phospho-ERK1/2, anti-ERK1/2, anti-phospho-JNK1/2, anti-JNK1/2, anti-phospho-p38, anti-p38, anti-acetyl-H3, anti-GAPDH, and HRP-conjugated secondary antibodies (Cell Signaling Technology); anti-HDAC1, anti-HDAC2 (Affinity BioReagents). All other chemicals were purchased from Sigma.

### Cell Culture

Human umbilical endothelial cells (HUVECs) (Sciencell) were cultured in endothelial cell medium (ECM) supplemented with endothelial cell growth supplement (ECGS), 5% fetal bovine serum (FBS), and penicillin/streptomycin (P/S) solution (Sciencell) in an incubator with 37°C, 5% CO_2_. For experiments, cells of passage no. 4–6 were grown until confluence. In some experiments, HUVECs were pretreated with SalA for 30 min and then subjected to the IS/RP protocol.

### Stimulated Ischemia/reperfusion (IS/RP) Experimental Protocol

HUVECs were subjected to oxygen–glucose deprivation (OGD) injury [40], [41]. The OGD injury was initiated by placing cells in a tri-gas incubator with an atmosphere of 37°C, 5% CO_2_, 94% N2, and 1% O2, and in the presence of OGD media for 5 h. After that, the exposure medium was replaced with oxygenated ECM, and the cells were placed in an incubator at 37°C, 5% CO_2_, to mimic the reperfusion conditions at different time points as indicated.

### siRNA Experiments

HUVECs were transfected with 50 nmol/L MKP-3 siRNA or nontargeting siRNA obtained from Dharmacon as described previously [16]. The nontargeting siRNA was used as transfection control for non-sequence-specific effects of the transfected siRNAs.

### Plasmids and Transfection

The HA-tagged constitutively active ERK1 and c-Myc-tagged constitutively active ERK2 for expression in mammalian cells were constructed by polymerase chain reaction (PCR), followed by cloning into the pcDNA3.1 plasmid (Invitrogen). HUVECs (60% confluence) were transfected using the lipofectamine 2000 (Invitrogen). Cells were incubated with the DNA-lipofectamine complexes at 37°C for 4 h followed by recovery in the presence of 0.5% FBS. Transfection efficiency was about 70% as determined using green fluorescent protein and maximal levels of protein expression were observed between 24 and 48 hours.

### Quantitative Real-time RT-PCR Analysis

Total RNA was purified from HUVECs with Trizol (Invitrogen). Real-time PCR was performed using Bio-RAD iQ5 Multicolor Real-Time PCR Detection System with SYBR Green as fluorescent and ROX (Takara) as reference dyes as described previously [42]. The specific primers used were as follows: human MKP-3∶5′-CTTGGTACATTGCTTGGCTGGCAT-3′ (forward), 5′-AAGAGAAACTGCTGAAGGGCCAGA-3′ (reverse); human eNOS: 5′-GCGGCTGCATGACATTGAG-3′ (forward), 5′-TCGCGGTAGAGATGGTCAAGTCA-3′ (reverse); human GAPDH: 5′-GACCCCTTCATTGACCTC-3′ (forward), 5′-GCTAAGCAGTTGGTGGTG-3′ (reverse).

### Immunoblot Analysis

Immunoblotting was performed according to the previous protocols [43]. Total proteins were extracted from HUVECs using cell lysis buffer (50 mM Tris-Hcl [PH 7.5], 150 mM NaCL, 0.5% NP40, 0.5% Triton-X100, 2 mM EDTA, 1 mM NaF, 1 mM PMSF, 1 mM Na_3_VO_4_, and Protease inhibitor cocktail (Roche)). Protein (40–60 µg) from each sample were separated by SDS-PAGE, transferred to nitrocellulose membranes, and probed with primary antibodies and then with horseradish peroxidase-conjugated secondary antibodies. The enhanced chemiluminescence signal was quantified using a densitometry program (Gel-pro 4.5 Analyzer, Media Cybernetics). To quantify the protein signal, we subtracted background, normalized the value to GAPDH. As for the phospho-specific protein, we normalized the signal to the amount of total target protein and GAPDH.

### Measurement of NOS Activity

The conversion of [3H]L-arginine to [3H]L-citrulline was used to assess eNOS activity in cultured endothelial cells using the NOS Activity Assay Kit (Cayman Chemicals, Inc. USA) as described previously [44]. Briefly, cultured HUVECs were suspended by brief treatment with trypsin–EDTA and washed with PBS. To prepare total cell lysates, cells were then suspended in cold lysis buffer at 4°C for 1 hour and centrifuged at 10 000 g for 20 minutes. Protein concentrations were determined using the BCA Protein Assay (Pierce, USA). To analyze eNOS activity, cell lysates (150 mg) were incubated with 1 mM NADPH, 0.6 mM CaCl2, 100 nM calmodulin, [3H]L-arginine (58 Ci/mmol, 1 mCi/mL), 1 mM flavin adenine dinucleotide, 1 mM flavin adenine mononucleotide, and 3 mM BH4 for 15 minutes at 37°C. In all experiments, replicate incubations were performed in the presence of the NOS inhibitor 1 mM L-NNA. Incubations were terminated by adding 0.4 mL of ice-cold stop buffer. [3H]L-arginine was separated from [3H]L-citrulline by passing the entire reaction mixture over 0.1 mL of equilibrated Dowex AG 50 WX-8 cation exchange resin (Bio-Rad Laboratories,USA). The effluent containing [3H]L-citrulline was collected and quantified by liquid scintillation counting. The activities were expressed as pmol citrulline/mg protein/min.

### Quantification of NO Formation in Cells

NO formation was detected as nitrite released into supernatant of confluent cultures of cells. Released nitrite was measured using the Griess colorimetric reaction [45]. This reagent reacts with nitrite in the medium to yield an Azo compound which has an absorption band at 540 nm. Triplicate samples of supernatant from endothelial cells were transferred to a 96-well ELISA plate, and an equal volume of the Griess reagent was added to each well. After 10 minutes of incubation at 37°C, the absorption at 540 nm was measured using an ELISA reader. A standard curve was constructed using known concentrations of sodium nitrite over the linear range of the assay.

### Evaluation of Apoptosis

Apoptosis was analysed by In situ Cell Death Detection Kit (Roche) according to manufacturer’s instructions [46]. Cells were observed by a Nikon microscope (ECLIPSE TE2000-U) with a 20X/0.45 NA air objective lens, and images were captured using a Nikon digital camera DXM1200 F and ACT-1 software (Nikon). The number of TUNEL-positive cells was quantified, and at least 120 cells per dish were counted.

### HDAC Activity Assay

HDAC activity was determined using the fluorometric HDAC activity assay kit (BioVision Inc.). Nuclear extracts (10 µg) was obtained from the cells, and the t-butoxycarbonyl-Lys(AC)-amido-4-methylcoumarin substrate (Boc-Lys(AC)AMC) was added, then, the incubation was performed at 37°C for 30 min. After incubation, the lysine developer was added, and the mixture was incubated again for 30 min at 37°C. Fluorescence was detected using a Spectra Max M5 fluorescent plate reader (Molecular Devices), with excitation at 360 nm and emission at 460 nm. All reactions were conducted in duplicate.

### Chromatin Immunoprecipitation Assay

The chromatin immunoprecipitation assay (ChIP) was performed as described previously [33], [47]. In brief, HUVECs were cross-linked with 1% formaldehyde at 37°C for 10 min at room temperature. The cell lysate was sonicated at 30% output on ice four times with 15-s intervals and pre-cleared with protein A-Sepharose 4B (Amersham Biosciences). The lysate was precipitated with the primary antibodies to acetylated histone 3 (Ac-H3, 3 µg) or HDAC1 (4 µg) or HDAC2 (4 µg) overnight at 4°C. The DNA was purified using the QIA-AMP DNA purification system (Qiagen). PCR products were detected, using primers specific for human eNOS eNOS (−238/−13), forward primer: 5′-AGC CCT GGC CTT TTC CTT AG-3′, reverse primer: 5′-GCT CCC ACT TAT CAG CCT CA-3′. Equal input DNA control was assessed and anti-Flag antibody (Sigma) was served as the negative control.

### Statistical Analysis

Each experiment was performed at least in triplicate. Data are presented as means±SEM. Differences between groups were evaluated for statistical significance using Student’s *t*-or ANOVA test [48]–[51]. In each case, significance was defined as *P*<0.05.
